# Time-domain diffuse correlation spectroscopy (TD-DCS) for noninvasive, depth-dependent blood flow quantification in human tissue in vivo

**DOI:** 10.1038/s41598-021-81448-5

**Published:** 2021-01-19

**Authors:** Saeed Samaei, Piotr Sawosz, Michał Kacprzak, Żanna Pastuszak, Dawid Borycki, Adam Liebert

**Affiliations:** 1 Nałęcz Institute of Biocybernetics and Biomedical Engineering, Polish Academy of Sciences, Ks. Trojdena 4, 02-109 Warsaw, Poland; 2 Institute of Physical Chemistry, Polish Academy of Sciences, Kasprzaka 44/52, 01-224 Warsaw, Poland; 3 Department of Neurosurgery, Mossakowski Medical Research Center Polish Academy of Sciences, Warsaw, Poland

**Keywords:** Imaging and sensing, Optics and photonics, Near-infrared spectroscopy

## Abstract

Monitoring of human tissue hemodynamics is invaluable in clinics as the proper blood flow regulates cellular-level metabolism. Time-domain diffuse correlation spectroscopy (TD-DCS) enables noninvasive blood flow measurements by analyzing temporal intensity fluctuations of the scattered light. With time-of-flight (TOF) resolution, TD-DCS should decompose the blood flow at different sample depths. For example, in the human head, it allows us to distinguish blood flows in the scalp, skull, or cortex. However, the tissues are typically polydisperse. So photons with a similar TOF can be scattered from structures that move at different speeds. Here, we introduce a novel approach that takes this problem into account and allows us to quantify the TOF-resolved blood flow of human tissue accurately. We apply this approach to monitor the blood flow index in the human forearm in vivo during the cuff occlusion challenge. We detect depth-dependent reactive hyperemia. Finally, we applied a controllable pressure to the human forehead in vivo to demonstrate that our approach can separate superficial from the deep blood flow. Our results can be beneficial for neuroimaging sensing applications that require short interoptode separation.

## Introduction

The blood flow is responsible for distributing nutrients and removing metabolic waste products. Any disorders in blood flow can lead to severe diseases or injuries. Thus, noninvasive modalities for perfusion measurements play a critical role at the clinical sites, especially in monitoring patients with cerebral blood flow (CBF) impairments. A variety of technologies for noninvasive blood flow measurement were developed. On the one hand, methods like ultrasound are sensitive to large blood vessels^1^. On the other hand, the approaches such as laser Doppler^2^, color Doppler optical coherence tomography^3^, laser speckle imaging^4^, and thermal methods^5^ can be used to monitor the blood flow below the tissue surface. However noninvasive CBF monitoring requires sensitivity to microvascular blood flow in deep tissue layers. One promising way to estimate CBF is through diffuse correlation spectroscopy (DCS)^6^. In DCS, coherent near-infrared light illuminates the tissue, and moving scattering particles (red blood cells) generate fluctuations of intensity (or more generally optical field), recorded at a distance $\rho$ from the emitter. DCS was successfully employed to quantify blood flow in many in vivo studies and has several promising applications in neuroimaging.

However, in DCS the blood flow is integrated over all photon paths. Consequently, the desired, cortical signal is confounded by photons traversing the skull and scalp without reaching the cortex. Usually, this problem is compensated for by increasing source-detector distance (SDS) to 2–3 cm. This improves sensitivity to deep tissue layers^7^ but reduces the detected signal intensity. According to diffusion theory, the number of diffusively reflected photons decreases exponentially with the square of the SDS^8^. Ideally, we want to keep SDS short ($\le$ 1 cm) to improve spatial resolution^9^, but decreasing SDS increases blood flow estimation errors^10^.

Potentially, the above issues could be solved by supplementing DCS with a mechanism for probing optical field fluctuations with a time-of-flight (TOF) resolution. Changes in blood flow in deep tissue (long TOFs) would then be quantified independently on the changes appearing in superficial regions (short TOFs). The pioneering contribution in this area comes from Yodh et al., who introduced pulsed diffusing wave spectroscopy (PDWS)^11^. In PDWS, the TOF-resolved field fluctuations are sensed with the nonlinear optical mixing through the second-harmonic generation. Interferometric near-infrared spectroscopy (iNIRS) also quantifies TOF-resolved dynamics in the turbid media^12^, rodent brain in vivo^13^, and humans in vivo^14^ through Fourier-domain interferometric detection.

Another promising approach is the time-domain (TD-) DCS^9,15–18^. Sutin et al. introduced this technique and utilized it to probe cerebral blood flow in rodent brains at short $\text {SDS}<1$ cm^15^. Pagliazzi and others implemented TD-DCS using high coherence laser^16^, and measured the relative blood flow index (rBFI) in the human forearm during the cuff occlusion challenge at $\text {SDS}=1$ cm^16^ and quasi-null SDS^17^. Tamborini et al. demonstrated the portable version of the TD-DCS system^9^, while Colombo et al. developed TD-DCS above the water absorption peak^18^.

**Figure 1:**
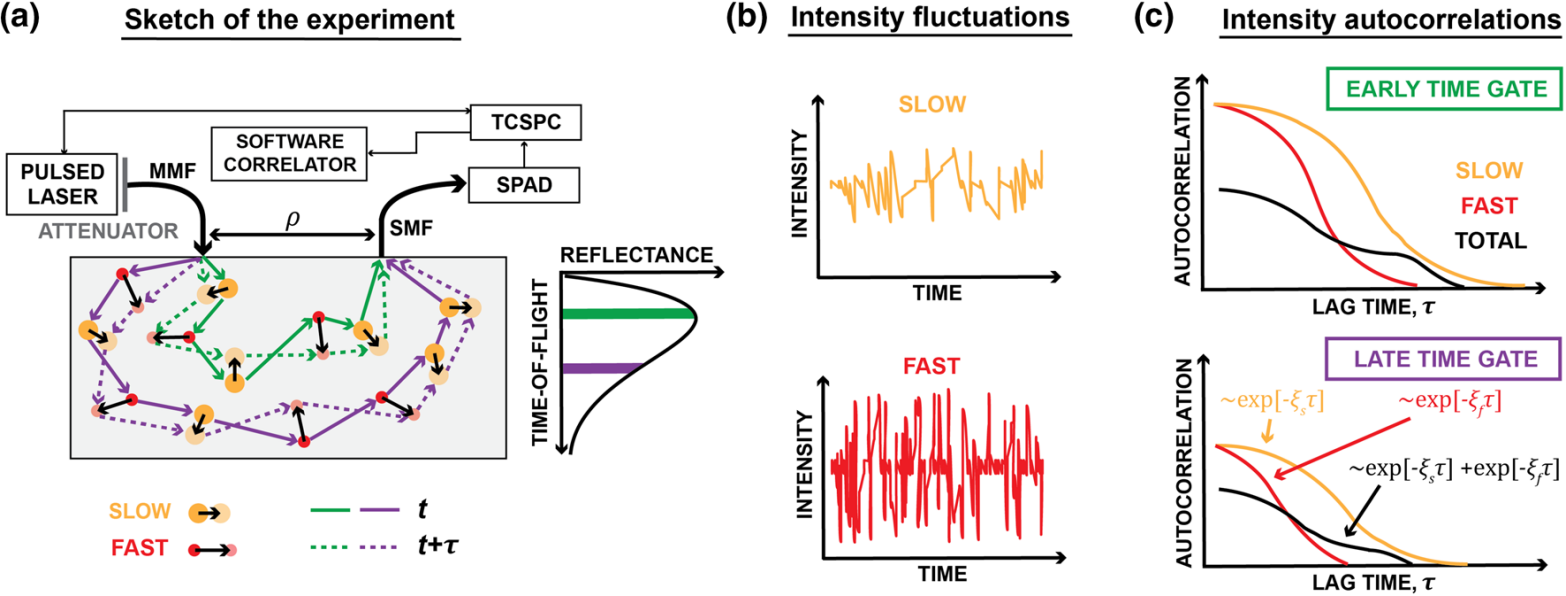
Time-domain diffuse correlation spectroscopy in samples composed of particles moving at different speeds. (**a**) The light emerging from the source is multiply scattered from slowly (orange), and rapidly (red) moving particles inside the sample, and then reaches the detector. As the scattering particles move, the photon light paths change over time (solid and dashed lines), leading to intensity fluctuations at the detector (**b**). (**c**) The larger the particle speed, the more rapid intensity fluctuations. Thus, the intensity autocorrelation function (ACF) of the rapidly moving particles decays faster (red line) than that of slow particles (orange line). However, the total ACF (black line) contains contributions from all photons. We disentangle these contributions using a multi exponential fitting. As the time-of-flight increases (late time gate), we register lower number of correlated photons. Hence, the intensity autocorrelation contrast decreases.

In TD-DCS, the light from a pulsed laser is injected into the sample through the multi-mode fiber (MMF), and the diffusively reflected light is collected with the single-mode fiber (SMF), and detected with a single-photon avalanche detector (SPAD) (Fig. 1). Similarly as in the time-domain near-infrared spectroscopy (TD-NIRS)^19,20^, the detected photons are time-tagged with time-correlated single-photon counting (TCSPC). However, unlike TD-NIRS, TD-DCS uses two tags. The first tag corresponds to the time needed for the photon to travel from the source to the detector, and is used to estimate the photon time-of-flight (TOF). The second tag corresponds to the absolute arrival time since the measurement was started, and is employed to determine intensity autocorrelation function (intensity ACF). Such a two-dimensional gating allows quantifying BFI with the TOF resolution through the TOF-resolved intensity ACF, commonly defined as^15^:

1 $$\begin{aligned} g_2(t_{s},\tau ) = \left<I(t_{s},t)I(t_{s},t+\tau )\right>_{t} / \left<I(t_{s},t)\right>^2_{t}, \end{aligned}$$

where $t_{s}$ denotes the time-of-flight, $\tau$ is the autocorrelation lag time, and $\left<\ldots \right>_{t}$ denotes temporal averaging with respect to *t* distinct from $t_{s}$.

In most of the TD-DCS studies the measured time-of-flight-resolved intensity autocorrelation function, $\hat{g}_2(t_{s}, \tau )$ is fit to the following model (Siegert relationship)^15^:

2 $$\begin{aligned} g_{2}^{(1)}(t_{s}, \tau ) = 1 + \beta |g_{1}^{(1)} (t_{s}, \tau )|^2, \end{aligned}$$

where $g_{1}^{(1)} (t_{s}, \tau ) = \exp \left[ -\xi (t_{s})\tau \right]$ is the single exponential optical field autocorrelation function. The symbol $\xi (t_{s})$ stands for the TOF-dependent ACF decay, whose particular form depends on the underlying model for the scatterers movement, and $\beta$ is the intensity ACF contrast^21^.

The above approach presumes that all scatterers move, on average, at the same speed. However, this assumption holds well only for homogeneous samples. On the contrary, biological organs comprise tissues of different kinds, including epithelial (skin), adipose, and muscle tissues. Due to the difference between the metabolism of each layer^22^, cells in those tissues can move at different speeds^7^. This effect impacts the DCS and TD-DCS because the light injected to the organ through the skin is scattered by different particles before reaching the detector (Fig. 1a). The detected intensity of such multiply-scattered light contains contributions from all scattering events. To accurately quantify the blood flow, we need to resolve those contributions. It is thus reasonable to postulate that the field ACF is a convex sum:

3 $$\begin{aligned} g_{1}^{(M)} (t_{s}, \tau ) = \sum _{m=1}^{M}a_m\exp \left[ -\xi _{m}(t_{s})\tau \right] , \quad \sum _{m=1}^{M}a_m=1. \end{aligned}$$

We now have *M* exponential terms with distinct, TOF-resolved decays $\xi _{m}(t_{s})$. Each term corresponds to optical fields associated with photons scattered from particles moving at different speeds over the experimental time scale, $\tau$. The summation in Eq. (3) reflects the fact that different scatterers move independently. Hence, the corresponding scattered fields $U_{m}(t_{s},\tau )$ are uncorrelated with each other, which formally is expressed as $\left<U_{m}(t_{s},t)U_{n}(t_{s},t+\tau )\right>_{t} = 0$ for $m \ne n$.

Our approach extends previous studies, in which turbid samples are envisioned as a composition of static and dynamic particles^23,24^. Based on this, a phenomenological model, that uses a sum of two negative exponentials (for slow and fast scatterers) and a constant offset (for static particles) was used to estimate cerebral blood flow with iNIRS^14^. Here, by following the concept from the laser Doppler flowmetry to resolve particle speed distribution in the sample^25^, we use the general form of $g_{1}^{(M)} (t_{s}, \tau )$ comprised of *M* components. We then, substitute Eq. (3) into Eq. (2) to obtain the novel model for TD-DCS:

4 $$\begin{aligned} g_{2}^{(M)}(t_{s}, \tau )&= 1 + \beta |g_{1}^{(M)} (t_{s}, \tau )|^2 \\&= 1+ \beta \left| \sum _{m=1}^{M}a_m\exp \left[ -\xi _{m}(t_{s})\tau \right] \right| ^{2}. \end{aligned}$$

Finally, we fit $g_{2}^{(M)}(t_{s}, \tau )$ to the experimentally estimated $\hat{g}_{2}(t_{s}, \tau )$ from the TD-DCS setup sketched in Fig. 1a. This fitting yields TOF-resolved decays $\xi _{m}(t_{s})$, from which we obtain either diffusion coefficients, $\alpha D_{B,m}$ in phantoms or blood flow index in human tissue. In general, we have *M* decays. We usually interpret the largest decay as the one related to scatterers located deep into the sample. Photons scattered from the deeply located scatterers experience many scattering events, and thus decorrelate faster, which is associated with larger $\xi _{m}(t_{s})$.

Our approach differs from previous theoretical works. Recently, Li et al. introduced an analytical model for TD-DCS applied to multi-layer heterogeneous turbid samples^26^. In their approach $g_{1}^{(M)}(t_s, \tau )$ is represented as a product of several negative exponential functions:

5 $$\begin{aligned} g_{1}^{(M)}(t_s, \tau ) = \prod _{m=1}^{M} \exp \left[ -\xi _{m}(t_{s})\tau \right] = \exp \left[ -\sum _{m=1}^{M}\xi _{m}(t_{s})\tau \right] . \end{aligned}$$

However, with the above equation we cannot explain experimental observations. In fact, the experimentally estimated $\hat{g}_{1}$ from measured $\hat{g}_{2}$ [Eq. (2)] obtained independently by different researchers using various optical systems can have an offset from static scatterrers^13^ or exhibit a bi-exponential decay^10,14,16,27^. Thus, deviating from the single-exponential decay, predicted by Eq. (5). On the contrary, we validated our approach against measurements in tissue-mimicking phantoms and humans in vivo, and found that our model fits experimental data very well.

We also note that measured intensity autocorrelation functions, $\hat{g}_{2}(t_{s}, \tau )$ depends on the instrument response function or the IRF^28,29^. Then, to improve the signal-to-noise ratio, the experimental estimates are integrated over the photon TOF range, called the time gate. Consequently, the theoretical model can be extended to include both IRF and the normalized photon distribution of time-of-flight (DTOF) as we demonstrate under methods. Here, we employ Eq. (4) since we only consider narrow time gates, neglecting the IRF and TOF integration effects.

## Results

### Tissue-mimicking phantoms

To validate the feasibility of separating different flows in turbid media, we performed TD-DCS measurements in liquid phantoms. Each measurement was repeated $N=5$ times. First, we carried out the measurements on three homogeneous phantoms with the same absorption coefficient ($\mu _a = 0.06\,{\text{cm}}^{-1}$) and the variable reduced scattering ($\mu _s^{\prime } = 7.5, 10.0,$ and $12.5\,{\text{cm}}^{-1}$).

**Figure 2:**
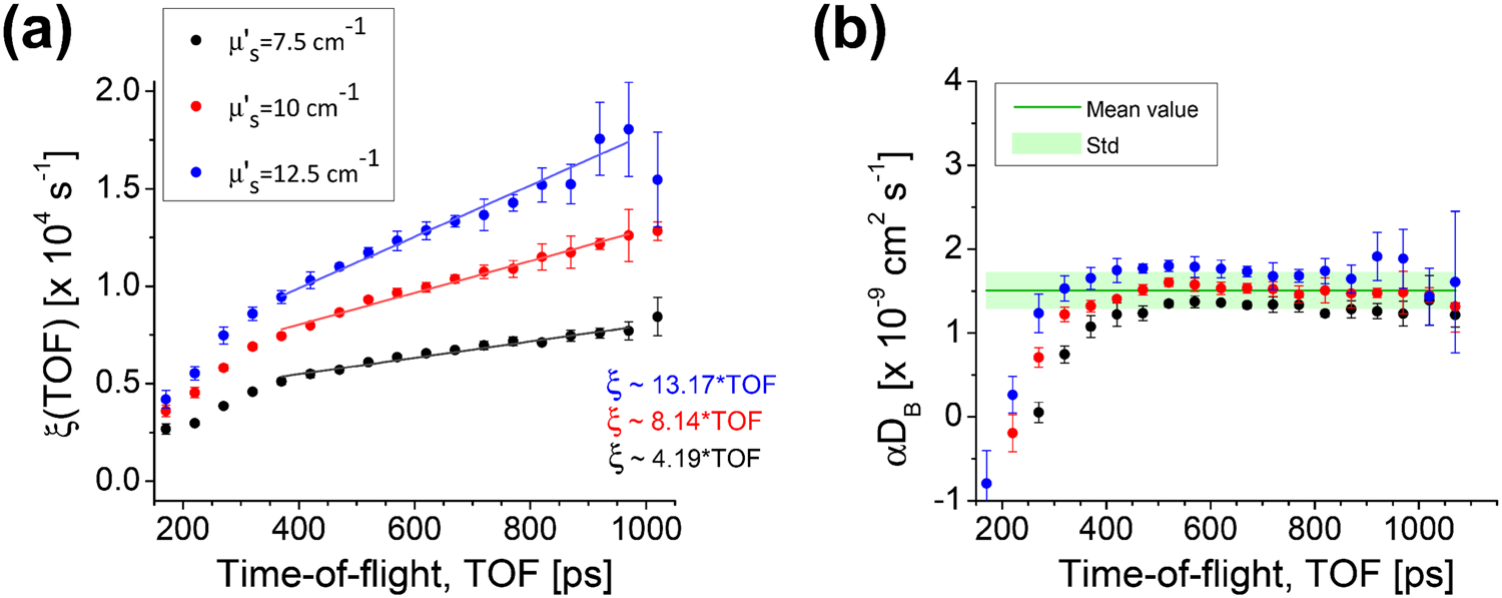
Estimating the diffusion coefficient of homogeneous phantoms with the time-domain diffuse correlation spectroscopy. (**a**) The autocorrelation decays were obtained using the standard model for the variable time-of-flight (TOF). The resulting decays were fitted to a line, from which we estimate the diffusion coefficient (**b**).

The TOF-resolved intensity ACFs were estimated using a 100 ps width time gate. Then by fitting the standard model to each ACF curve, we obtain the TOF-resolved autocorrelation decays $\xi (t_{s})$ (Fig. 2a), from which we calculate the diffusion coefficients, $\alpha D_{B}({\text {TOF}})$ [see Eq. (9) in Methods]. Figure 2b shows that resulting values of $\alpha D_{B}({\text {TOF}})$ are consistent across phantoms. Lastly, we averaged diffusion coefficients for TOF > 370 ps ($\alpha D_{B} = 1.51 \times 10^{-9}\,{\text{cm}}^{2}{\text{s}}^{-1}$), and then use it as the control value for the two-layer liquid phantoms.

The two-layer liquid phantoms were prepared such that the optical properties were kept constant in both layers, and we reduced the dynamical properties of the top layer. To do so, we mixed the liquid with glycerol (30% concentration) and controlled optical properties using recipe from Supplementary Fig. S2. The instrument response function (IRF) and the photon distribution of time-of-flight (DTOF) of the two-layer liquid phantoms are shown in Supplementary Video S1.

**Figure 3:**
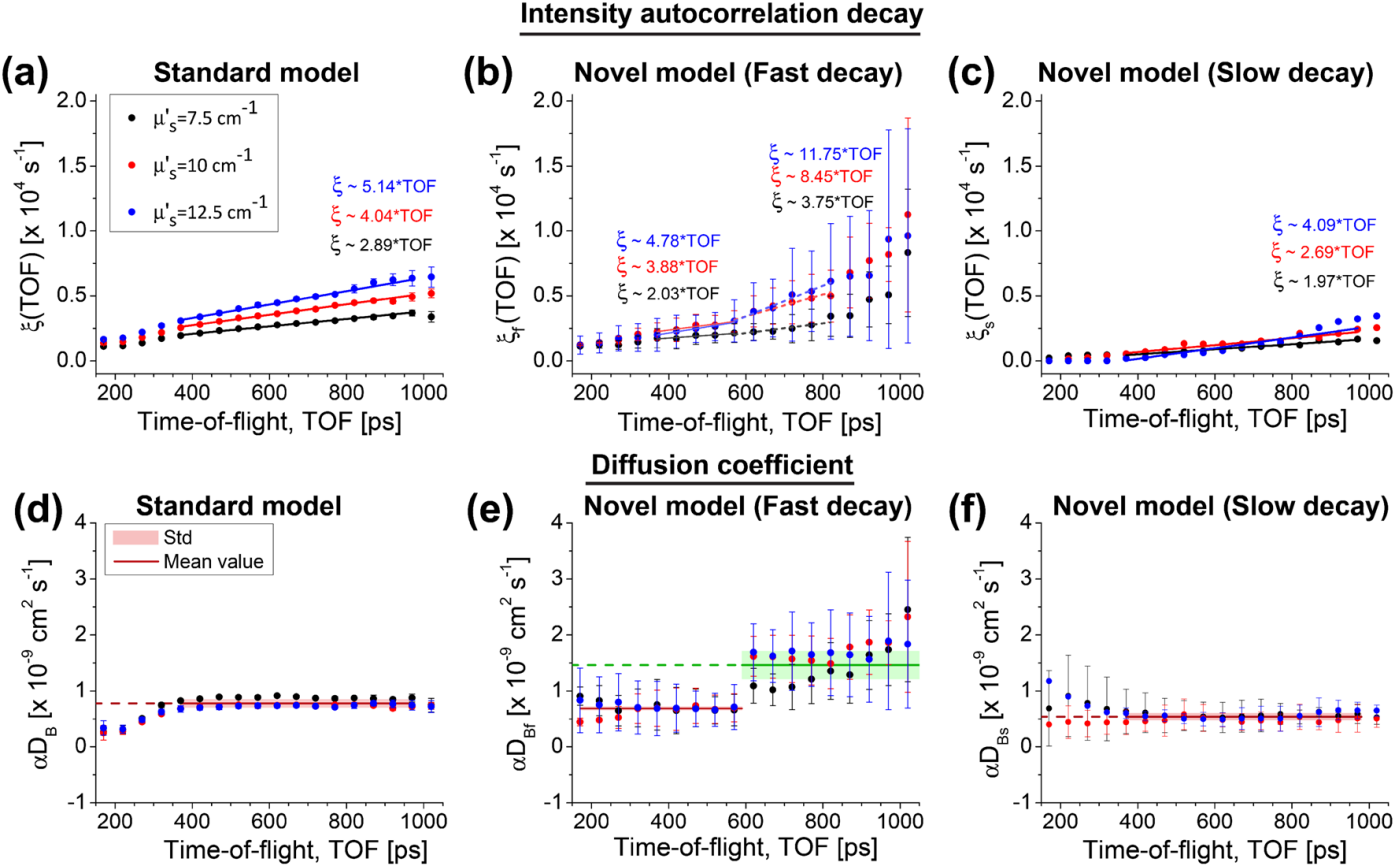
Time-domain diffuse correlation spectroscopy measurement in two-layer liquid phantoms with absorption coefficient ($\mu _a = 0.06\,{{\text{cm}}}^{-1}$) and variable reduced scattering ($\mu _s^{\prime } = 7.5, 10.0,$ and $12.5\,{{\text{cm}}}^{-1}$). (**a**–**c**) Estimated TOF-resolved autocorrelation decays and the diffusion coefficients (**d**–**f**). The standard model provides a constant slope of the ACF decays, and thus is incapable to distinguish flows in different layers (**d**). On contrary, the fast component of the novel model changes at 600 ps (**b**), which allows to clearly separate the depth-dependent diffusion coefficients (**e**). The slow component of the novel model senses the top layer along different TOFs by providing a uniform slope of ACF decay (**c**), and a constant diffusion coefficient (**f**) close to the value estimated by the fast component at early TOFs (**e**).

Subsequently, we estimated TOF-resolved intensity ACF using a time gate of 100 ps width. Experimental estimates were then fit with standard TD-DCS model [Eq. (2)] and novel model [Eq. (4)] with $M=2$ exponential ACFs. The representative fits are depicted in Supplementary Video S1 online. The fitting yielded TOF-resolved ACF decays, shown in Fig. 3a–c. Now, the novel model provides two ACF decays: fast $\xi _{f}(t_{s})$ (Fig. 3b) and slow $\xi _{s}(t_{s})$ (Fig. 3c). These components are used to determine the corresponding diffusion coefficients for each model (Fig. 3d–f).

Figure 3b shows that fast component, $\xi _{f}(t_{s})$ increases more rapidly for time-of-flights larger than 600 ps. Consequently, at this transition point, we observe larger values of $\alpha D_{B, f}$ since late photons probe deep phantom layer with faster particles. Last, the mean diffusion coefficient ($\alpha D_{B, f} = 1.46 \times 10^{-9}\,{\text{cm}}^{2}$s$^{-1}$) estimated for the late time gates ($570-820$ ps) using the fast component [$\xi _{f}(t_{s})$] of the novel model is almost the same as that of the homogeneous phantom ($\alpha D_{B} = 1.51 \times 10^{-9}\,{\text{cm}}^{2}$s$^{-1}$), indicated in Fig. 2b. Furthermore, the diffusion coefficient obtained from the slow component [$\xi _{s}(t_{s})$] of the novel model ($\alpha D_{B, s} = 5.40 \times 10^{-10}\,{\text{cm}}^{2}$s$^{-1}$) (Fig. 3f) is close to the values estimated for the time-of-flight earlier than 600 ps ($\alpha D_{B, f} = 6.88 \times 10^{-10}\,{\text{cm}}^{2}{\text{s}}^{-1}$) (Fig. 3e). On the contrary, the standard model underestimates diffusion coefficient ($\alpha D_{B} = 7.76 \times 10^{-10}\,{\text{cm}}^{2}{\text{s}}^{-1}$) due to the influence of the superficial layer.

### Human forearm in vivo

**Figure 4:**
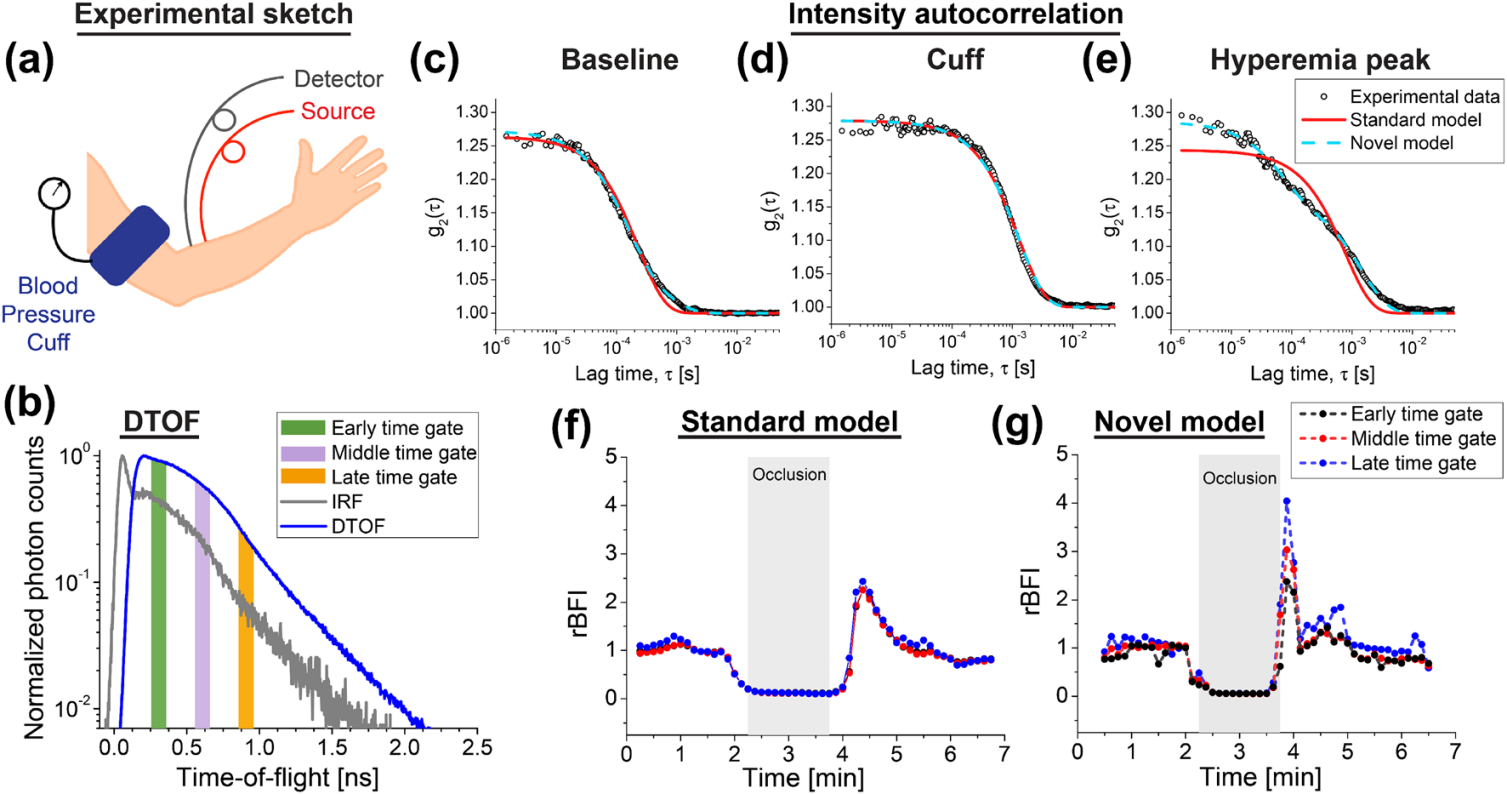
In vivo cuff occlusion challenge in human forearm. (**a**) Experimental sketch. (**b**) Representative DTOF, showing the time gates, used for estimating the relative BFI trends (**f**,**g**). (**c**–**e**) Representative intensity ACFs of different stages of the measurement. At the baseline, intensity ACF decorrelates much faster (**c**) than at the cuff occlusion stage (**d**). Right after the cuff is released, the intensity ACF is composed of the two ACFs that decorrelate at different rates (**e**). The novel model depicts the depth-dependent reactive hyperemia (**g**), while conventional TD-DCS model does not (**f**).

Next, we applied TD-DCS to measure blood flow in the cuff occlusion challenge on the left arm of three healthy volunteers in vivo (Fig. 4a). The representative DTOF is depicted in Fig. 4b. We estimated intensity ACFs at three different gates, and then fit them using standard and novel models (Fig. 4c–e).

Figure 4c,d shows that the intensity autocorrelation function decorrelates faster at the baseline stage than during the cuff was occluded. Furthermore, Fig. 4e clearly illustrates the influence of the mixture of slow and fast blood flows on the ACF just after the cuff deflation. In this case, the ACF decorrelates at different rates, and the novel model fits very well to, while the standard model deviates from the experimental data.

To quantify blood flow in muscle tissue, we only use the fast decay (because it provides the information about the rapidly moving scatterers, i.e. blood cells). However, now we interpret the diffusion coefficient as the blood flow index (BFI). Then, we calculated the relative blood flow index (rBFI) by normalizing the BFIs to the baseline. To obtain the baseline we averaged datapoints within the 60 s just before the cuff occlusion was applied.

The resulting rBFI trends for the standard and novel models are averaged over all three subjects and depicted in Fig. 4f,g, respectively. The results obtained for each subject are provided in Supplementary Fig. S1 online. For the standard model, the rBFI is independent of the time gate. From previous experiments^7^, we expect that rBFI for late photons, after the cuff is released, provide larger values than that of the early photons (reactive hyperemia is stronger for deep tissue layers, i.e., muscles). However, when the standard model is used, the rBFI trends do not exhibit such a behavior. Even though the light paths are distinguished by their TOF, the standard model does not provide depth selectivity. This means that TOF is insufficient to quantify the blood flow at various tissue layers, and the use of our approach is warranted.

### Human forehead in vivo

**Figure 5:**
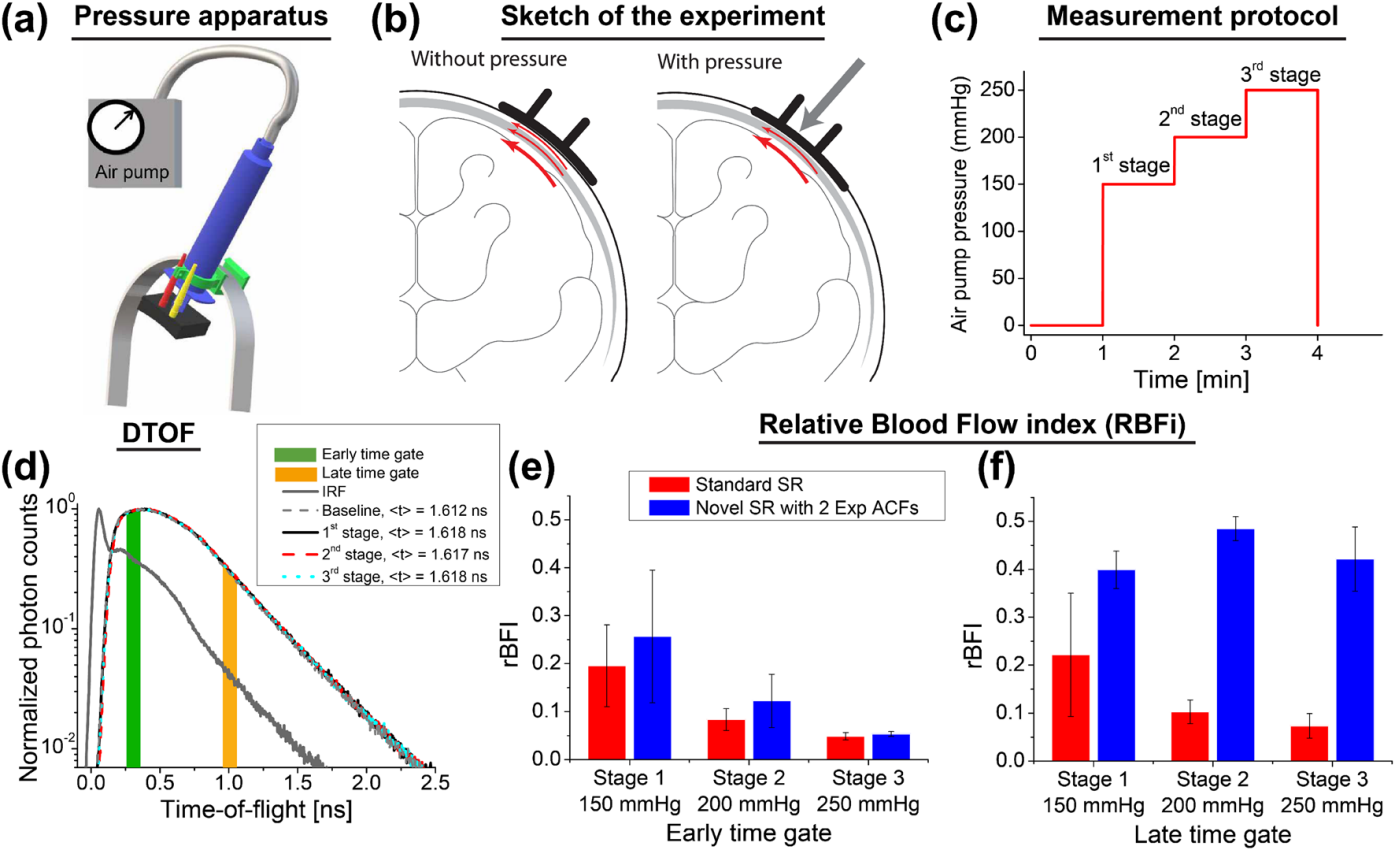
Measuring the relative blood flow index in the human forehead under variable pressure in vivo. (**a**) The sketch of the pressure apparatus (**a**), experiment (**b**), and tissue pressure protocol (**c**). (**d**) IRF and representative DTOFs for various stages of the experiment. Relative blood flow for early (**e**) and late time gate (**f**) are compared between the standard (red bars) and novel (blue bars) models.

Finally, we applied TD-DCS in the adult human forehead under controllable pressure on the forehead skin (Fig. 5). This experiment provides an illustrative example of how the static and slow scatterers located in extra-cerebral tissue (scalp or skull) contribute to the signal at short source-detector separation. By pressing the tissue, we change the superficial flow, while cerebral flow remains constant (Fig. 5b).

By analyzing DTOFs we confirmed that optical properties did not significantly change when the pressure value increased (Fig. 5d). Then, we estimated intensity ACFs, from which we calculated relative BFIs for early and late time gates (Fig. 5e,f). The BFIs were normalized to the baseline, measured before the pressure was applied. For the early gate (centered at 0.3 ns), the rBFI decreases with increasing pressure (Fig. 5e). However, we do not expect a similar behavior for the late time gates (centered at 1 ns). This is because applying pressure on the scalp blocks blood flow on superficial layers, but the skull prevents this pressure from being applied to the brain cortex. The rBFI derived from the novel model confirms this hypothesis (Fig. 5f), while the rBFI estimated from the standard decreases with increasing pressure. Though the novel model works as an additional gating mechanism, supplementing time gating, the two gates might not completely reject photons propagating extracerebral layers. This issue can also reduce the rBFI compared to the baseline value. Hence even for the novel model, the estimated rBFI is still below the baseline value. We expect that this problem can be solved by a more general fitting approach that includes the effect of IRF.

## Discussion and conclusions

In summary, we introduced and applied the novel model for time-domain diffuse correlation spectroscopy (TD-DCS), enabling independent quantification of various blood flows in human tissue. We demonstrated that sorting detected photons based on their penetration time in tissue by TD-DCS is insufficient to distinguish blood flows at different layers. To solve this problem, we applied our novel model, and quantified depth-resolved blood flows in heterogeneous media, particularly in human tissue. We validated our approach via measurements in tissue-mimicking phantoms, and the cuff occlusion challenge on the human forearm in vivo.

The diffusion coefficient of the homogeneous liquid phantoms were estimated by using the standard model, and the averaged value ($\alpha D_{B} = 1.51 \times 10^{-9}\,{\text{cm}}^{2}{\text{s}}^{-1}$) was considered as a reference. Next, in the two-layer liquid phantom measurements, we utilized the reference media in the deep layer covered with a liquid phantom comprising slower scatterers and matched optical properties. Then by employing the novel we quantified the diffusion coefficient of both layers. The fast component of the novel model recovered the reference value from the deep layer ($\alpha D_{Bf} = 1.46 \times 10^{-9}\,{\text{cm}}^{2}{\text{s}}^{-1}$), while the slow component estimated the diffusion coefficient of the top layer, mixed with 30 % glycerol, around 36 % lower than the reference value ($\alpha D_{Bs} = 5.40 \times 10^{-10}\,{\text{cm}}^{2}{\text{s}}^{-1}$), as expected^30^. Importantly, the standard model was incapable to distinguish variable dynamics in both layers and provided a constant value of $\alpha D_{B} = 7.76 \times 10^{-10}\,{\text{cm}}^{2}{\text{s}}^{-1}$.

We performed forearm cuff occlusion measurements on healthy adults in vivo. We detected the depth-resolved blood flow changes by using the fast component of the novel model. We showed a higher magnitude of hyperemic peak after the cuff pressure deflation corresponding to late time gate, which is due to higher hemodynamic changes in muscle than in adipose tissue^7^. Importantly, this was not possible with the standard model, which estimated a similar rBFI trend for early and late time gates. In fact, the magnitude of the hyperemia peak estimated by the standard model is close to the values measured from the novel model for the early time gate, which can be due to the long-tailed IRF. The relative rBFI changes for slow components and each subject is available in Supplementary Fig. S1 online.

Finally, we performed measurements on the forehead of an adult human volunteer under the variable pressure. By employing the novel model we reduced the effects of the superficial layer and obtained a constant level of deep layer blood flow, during various pressure stages. In order to distinguish the contribution of blood flowing in the skull from the cortex blood flow, further studies on multi-layers phantoms with a more general approach are required.

The optical setup suffers from the limited laser coherence length and detected photon count rates. We tackled these issues by utilizing short source-detector separation (1 cm) and increasing the recording time. On the one hand, by increasing collection time we reduce the time-resolution. Thus, we cannot detect fast fluctuations like pulsatile heart beat. On the other hand, by using short source-detector separations, the detected signals are significantly affected by superficial layers. By employing our novel processing method we minimize the contamination from those layers.

Virtually, we can use any number of exponentials [*M* parameter in Eq. (4)]. However, by increasing *M* we also increase the overall number of fitting parameters. Accordingly, the fitting becomes more complex and sensitive to numerical errors.

In summary, we expect our method to be applicable in scenarios that require short source-detector separations. For example, brain-computer or body-computer interfacing. Our processing can be applied to any method that is oriented at quantification of particles movement in scattering medium with scattered light correlations, including DCS, interferometric NIRS, interferometric diffusing wave spectroscopy (iDWS), and fluorescence correlation spectroscopy (FCS).

## Methods

### Optical system

In our TD-DCS system, we use an 80 MHz pulsed laser (LDH-P-C-N-760, PicoQuant), emitting pulses shorter than 90 ps at a wavelength, $\lambda$ of 760 nm. Using the commercial spectrometer we estimated the coherence length of this laser to 6.1 mm. Light from the laser is delivered to the sample surface through a 1 mm core diameter multi-mode fiber with a numerical aperture (NA) of 0.39. A variable neutral density attenuator was used to set the average optical power delivered to the surface of the medium to 12 mW. The diffusively reflected light is collected by a single-mode fiber, located at a distance $\rho$ from the source, and detected with a single-photon avalanche diode (SPAD) detector (PDM, Micro Photon Devices). The SPAD output is time-correlated with a reference signal from the laser controller using the time-correlated single-photon counting (TCSPC) module (SPC-130, Becker&Hickl). The TCSPC provides the time-of-flight and the absolute photon arrival time of each detected photon with the temporal resolutions of 3.5 ps (for the photon distribution of time-of-flight or DTOF) and 12.5 ns (for intensity autocorrelations), respectively.

The instrument response function (IRF), affecting the true DTOF, is measured by facing the source and detection fibers in front of each other. The tip of the detection fiber is covered with a sheet of white paper to fill up the full numerical aperture of the fiber^31^. By doing so, we estimated the IRF full width at half maximum (FWHM) to 100 ps. All measurements were performed in reflection geometry at source-detector separation, $\rho = 1$ cm. To ensure the conditions for all the experiments and minimize the environmental noise, the measurements were performed in a dark room at a temperature of about $25\,^{\circ }\hbox {C}$.

### Raw data processing

The output of the optical system (from TCSPC module) can be represented as a two-dimensional dataset, $N(t_{s},t)$. That is the number of photons, traveling from source to detector at a particular TOF with the absolute arrival time, *t*. We process TCSPC data to achieve the photon distribution of time-of-flight (DTOF) by integrating photon counts with the same TOFs:

6 $$\begin{aligned} \text {DTOF}(t_{s}) = \int _{0}^{T}N(t_{s}, t)dt, \end{aligned}$$

where *T* is the total measurement time. Photon counts $N(t_{s},t)$ are binned together within the time gate $T_{gw}$ of fixed width 100 ps, and converted to intensities:

7 $$\begin{aligned} \hat{I}(t_{s}, t) = \int _{-T_{gw}/2}^{T_{gw}/2} E_p N(t_{s} + t_{gw}, t)dt_{gw}, \end{aligned}$$

where $E_p = hc/\lambda$ is the single-photon energy (*h* is the Planck constant, and *c* stands for the speed of light in vacuum).

Then, we estimate intensity ACF as:

8 $$\begin{aligned} \hat{g}_{2}(t_{s}, \tau ) = \frac{\left<\hat{I}(t_{s}, t)\hat{I}(t_{s}, t+\tau )\right>_{t}}{\left<\hat{I}(t_{s}, t)\right>^{2}_{t}}. \end{aligned}$$

For all reported experiments, $\hat{g}_{2}(t_{s}, \tau )$ was obtained from a rectangular time gate with 100 ps width, which offers the best trade-off between the signal-to-noise ratio and the intercept of the normalized intensity autocorrelation function. Furthermore, the ACFs were estimated from the data recorded during 60 s, except the pressure-dependent measurements. Due to the employed protocol in the forehead pressure experiment, the integration time was reduced to 30 s to extract more than one data point without overlapping the neighbor stages at each part of the measurement.

### Estimating diffusion coefficient and blood flow index

To determine diffusion coefficient and blood flow index we proceed as follows. We fit $g_{2}^{(M)}(t_{s}, \tau )$ with variable *M* [Eqs. (3), (4)] to the experimentally estimated $\hat{g}_{2}(t_{s}, \tau )$ from the TD-DCS setup sketched in Fig. 1a. Using the Brownian motion model^6^, in which $\xi _m (t_{s}) \propto \mu _{s}^{\prime } \alpha D_{B,m} t_{s}$ ($\alpha$ is the parameter, that traditionally represents the fraction of dynamic to scattering events^6^, and $\mu _{s}^{\prime }$ is the reduced scattering), we estimate the diffusion coefficient ($\alpha D_{B_{m}}$) of the mth component.

In practice, the decay rates for short TOFs deviate from the Brownian motion model predictions. Therefore, we exclude those TOFs from further analysis, in which we fit the linear model $\xi _{m}(t_{s}) = p_{1} \times t_{s} + p_{0}$ [$s^{-1}$] to the experimentally estimated TOF-resolved ACF decays. The fitting procedure yields the slope, $p_1=2k^{2}\mu _{s}^{\prime } c \alpha D_{B,m} /n$ ($k=2\pi n/\lambda$, *n* is the refractive index, *c* denotes the speed of light in vacuum), and an offset, $p_0$. To obtain an accurate estimate of the $\alpha D_{B,m}$ we subtract an offset and calculate $\alpha D_{B,m}$ as:

9 $$\begin{aligned} \alpha D_{B,m}(t_{s}) = \frac{\left( \xi _m - p_0\right) n}{2k^{2}\mu _{s}^{\prime } c t_{s}}. \end{aligned}$$

When applying the above approach in tissue we call $\alpha D_{B,m}(t_{s})$ as the blood flow index (BFI).

### Phantom experiments

The measurements on fluid phantoms were performed in a custom-made cubic compartment with a side length of 6 cm. This chamber has black walls and simulates the semi-infinite geometry to satisfy light diffusion assumptions. The front plate of the compartment includes two tiny holes, with diameters of 2.5 mm, covered by a 23 $\mu$m thick transparent Mylar film to fix the fibers with 10 mm separation on the phantom surface.

First, we carried out the measurements on homogeneous liquid phantoms. The liquid tissue-mimicking phantoms were made by mixing homogenized milk (3.2 % fat), distilled water, and black ink (Rotring, Germany). The phantoms had the same absorption coefficient ($\mu _a=0.06\,{\text{cm}}^{-1}$), but differed in reduced scattering coefficients ($\mu _s^{\prime } = 7.5-12.5\,{\text{cm}}^{-1}$ in steps of $2.5\,{\text{cm}}^{-1}$). During the measurements on homogeneous phantoms, the compartment was uniformly filled by the phantoms. While, in order to perform measurements on two-layer liquid phantoms, a 23 $\mu$m thick Mylar sheet was fixed inside the compartment, parallel to the front plate, and with 0.5 cm separation from this plate, to separate the phantom layers. In each measurement, the optical properties of the liquids used in upper and deeper compartments were matched. The homogeneous phantoms were used in the deeper compartment, while the liquid in the upper part was mixed with glycerol (30% concentration) to slow down the scatterers^30^.

To tune the optical properties between the media, we first quantified the $\mu _s^{\prime }$ based on the concentrations of scattering component (milk) and glycerol [Supplementary Fig. S2]. Then we added black ink (Rotring) to increase the absorption coefficient of the phantom to $\mu _a=0.06\,{\text{cm}}^{-1}$ by using the recipe from^32^. The optical properties of each sample were controlled using a TD-NIRS setup and moment approach^19,33^, separately. This system was constructed using the same laser diode as in our TD-DCS instrument (operating at a wavelength of 760 nm) and a photomultiplier detector (PMC-100, Becker & Hickl) coupled with a multi-mode fiber (core diameter 600 $\upmu$m).

To obtain a similar signal-to-noise ratio across all the phantom measurements, the optical power of the source was controlled with the neutral density attenuator, located in front of the laser head (Fig. 1a). We tuned the count rate to 1362 Kcps for each measurement. The raw signals of each experiment were recorded in five repetitions with 1 min collection time.

### In vivo measurement protocols

We applied the TD-DCS method to quantify blood flow index (BFI) time courses during the cuff occlusion challenge in the human forearm and forehead pressure measurement on adult healthy volunteers in vivo. These measurements were performed at 1 cm source-detector separations and the optical power of the source delivered to the tissue surface was 12 mW. All experimental procedures and protocols were reviewed and approved by the Commission of Bioethics at the Military Institute of Medicine, Poland (permission no. 90/WIM/2018). The experiments were conducted following the tenets of the Declaration of Helsinki. Written informed consent was obtained from all subjects before TD-DCS sensing and explaining all possible risks related to the examination. The physiological parameters of the participants are given in the Supplementary Table S1 online.

To monitor blood flow changes during the cuff occlusion challenge, the source and detector fibers were fixed in a black 3D printed square fiber holder, with a side length of 6 cm. The fiber holder was secured over the *flexor carpi radialis* with an elastic bandage. The organ went through three different physiological stages. First, the rest state was measured for 2 min to determine the baseline blood flow index (BFI). Second, the blood pressure cuff was inflated quickly to 180 mmHg and was held for 2 min. Third, the cuff was released, and we measured the recovery state for 3 min. This measurement was carried out on three volunteers.

To apply a uniform and controllable pressure on the participant forehead, we developed a pressing mechanism, comprising a cylinder pumped by air at tunable pressure levels, and its connecting rod was attached to the probe mounting the optodes (Fig. 5a). The probe was a black 3D-printed panel which held source and detector fibers by 1 cm separation, and covered the tissue curvature. The measurement was carried out on one of the volunteers and repeated three times in the same day. The participant was asked to lay supine on a bed, and the probe placed on the subject’s scalp directly over the right prefrontal cortex. One minute rest started the experiment, and then the tissue was pressed in three stages with variable pressure: 150, 200, and 250 mmHg. Each pressure was applied for 1 min (Fig. 5c).

### Statistical analysis of the fitting

To quantify fitting with standard and novel models, we performed a statistical analysis of the sample fits we achieved for in vivo experiments (cuff occlusion challenge on participant C). We used the intensity autocorrelations estimated at the middle time gate (centered at the 0.57 ns). Then, we performed fitting for the standard and novel model and calculated the following statistical tests: the sum of squares (SSE), R-square, adjusted R-square, degree of freedom in error (DFE), and the root mean squared error of standard error (RMSE). The results are summarized in Table 1.

**Table 1:** Statistical analysis of the fitting with standard and novel model.

Model	Number of parameters	$R^{2}$	Adjusted $R^{2}$	DFE	SSE	RMSE
Novel	4	0.95	0.94	56	7.81E−3	1.18E−2
Standard	2	0.80	0.80	58	3.00E−2	2.28E−2

### General expression for the intensity autocorrelation function

The measured TOF-resolved intensity autocorrelation function $\hat{g}_{2}(t_{s}, \tau )$ depends on the instrument response function or the IRF, and the photon time-of-flight distribution, when experimental estimates are integrated over the TOF. Thus, under the validity of Eq. (2), we can include those effects as follows^28^:

10 $$\begin{aligned} g_{2}^{(M)}(t_{s}, \tau , T_{gw}) = 1 + \beta \left| \int _{-T_{gw}/2}^{T_{gw}/2}dt_{s}^{\prime }P^{\prime }(t_{s} + t_{s}^{\prime }) \sum _{m=1}^{M}a_m\exp \left[ -\xi _{m}(t_{s}+t_{s}^{\prime })\tau \right] \right| ^{2}, \end{aligned}$$

where $T_{gw}$ is the gate width, and $P^{\prime }(t_{s})$ is the normalized measured photon distribution of time-of-flight (DTOF), which is related to the true photon TOF distribution $P(t_{s})$ via a convolution ($\star$) with the IRF, $I_{0}(t_{s})$:

11 $$\begin{aligned} P^{\prime }(t_{s}) = \frac{1}{N_{P}} P(t_{s}) \star I_{0}(t_{s}), \end{aligned}$$

where $N_{P} = \int _{-\infty }^{\infty } dt_{s} P(t_{s}) \star I_{0}(t_{s})$ is the normalization factor.

## Supplementary information

Supplementary material 1

Supplementary material 2
