# Supplementary material for: Time-domain diffuse correlation spectroscopy (TD-DCS) for noninvasive, depth-dependent blood flow quantification in human tissue in vivo
Source: Sci Rep. 2021 Jan 19;11:1817. doi: 10.1038/s41598-021-81448-5 (PMC7815740; doi:10.1038/s41598-021-81448-5)
Supplement: Supplementary file 1 — Supplementary material 1 [file 41598_2021_81448_MOESM1_ESM.pdf]

Supplementary Material:

Time-domain diffuse correlation spectroscopy (TD-DCS) for noninvasive, depth-dependent blood flow quantification in human tissue *in vivo*

Saeed Samaei[1,2], Piotr Sawosz[1], Michał Kacprzak[1], Żanna Pastuszak[3], Dawid Borycki[2,*,+], and Adam Liebert[1,+]

[1]Nalecz Institute of Biocybernetics and Biomedical Engineering, Polish Academy of Sciences, Ks. Trojdena 4, 02-109, Warsaw, Poland
[2]Institute of Physical Chemistry, Polish Academy of Sciences, Kasprzaka 44/52, 01-224 Warsaw, Poland
[3]Department of Neurosurgery, Mossakowski Medical Research Center Polish Academy of Sciences, Warsaw, Poland [*]Corresponding author: dborycki@ichf.edu.pl
[+]These authors share senior authorship

Supplementary materials

Supplementary Video S1: This visualization shows instrument response function (IRF), distribution of photon time-of-flight (DTOF), and autocorrelation functions for variable time-of-flight in phantoms.

Supplementary Tables

Supplementary Table S 1. Physiological parameters of healthy volunteers participating in *in vivo* experiments.

Parameter	Subject A	Subject B	Subject C
Weight [kg]	52	53	70
Height [cm]	167	173	172
Age	31	29	26
Gender	F	M	M
BMI Index	18.6	17.7	23.7
Body fat percentage (BMI method)	24.8%	12.0%	18.2%
Forearm superficial layer thickness [mm]	1.95	1.25	2.05
Forehead scalp thickness [mm]	–	2.3	–

Supplementary Figures

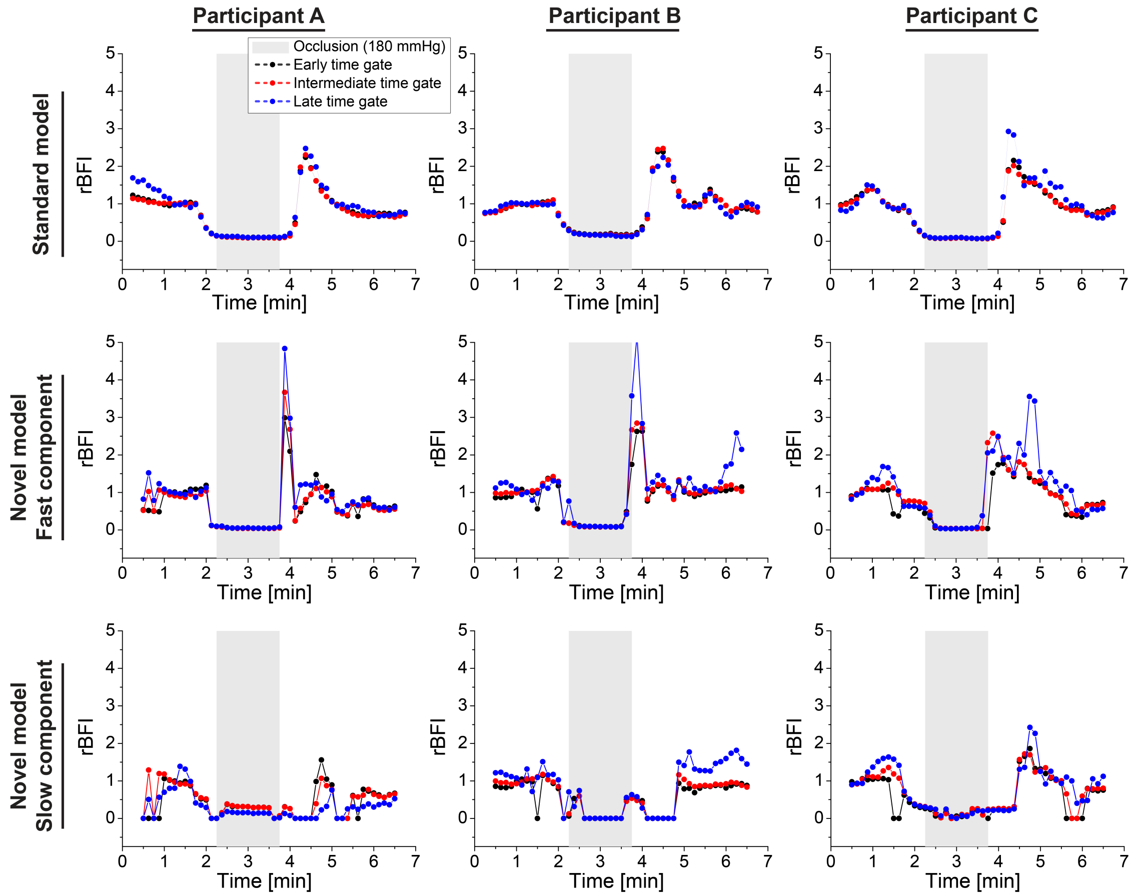

Supplementary Figure S 1. Relative blood flow index changes of each participant (columns) during the cuff occlusion challenge *in vivo*, obtained from the standard (top row) and novel model (middle and last row).

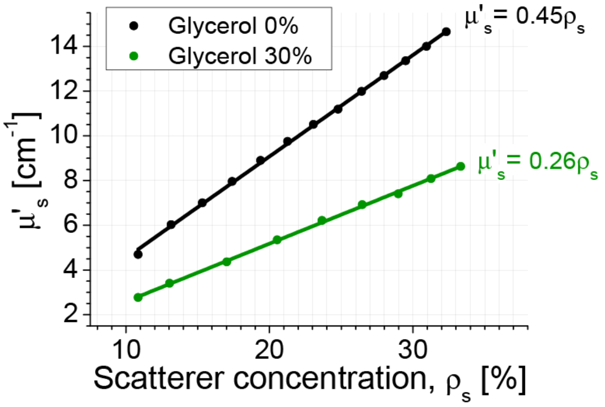

Supplementary Figure S 2. The reduced scattering coefficient, μ_s' at $\lambda = 760\ nm$ as a function of glycerol and scatterer concentration (milk 3.2 % fat) concentrations.
